# Neuroprotection of retinal ganglion cells by a novel gene therapy construct that achieves sustained enhancement of brain-derived neurotrophic factor/tropomyosin-related kinase receptor-B signaling

**DOI:** 10.1038/s41419-018-1041-8

**Published:** 2018-09-26

**Authors:** Andrew Osborne, Tasneem Z. Khatib, Lalana Songra, Amanda C. Barber, Katie Hall, George Y. X. Kong, Peter S. Widdowson, Keith R. Martin

**Affiliations:** 1 John van Geest Centre for Brain Repair, Department of Clinical Neurosciences, University of Cambridge, Cambridge, UK; 2 Quethera Ltd, Babraham Research Campus, Cambridge, UK; 3 Eye Department, Addenbrooke’s Hospital, Cambridge, UK; 4 Centre for Eye Research Australia, Royal Victorian Eye and Ear Hospital, Melbourne, Australia; 5 University of Melbourne, Melbourne, Australia; 6 Cambridge NIHR Biomedical Research Centre, Cambridge, UK; 7 Wellcome Trust—MRC Cambridge Stem Cell Institute, University of Cambridge, Cambridge, UK

## Abstract

Previous studies have demonstrated that intravitreal delivery of brain-derived neurotrophic factor (BDNF) by injection of recombinant protein or by gene therapy can alleviate retinal ganglion cell (RGC) loss after optic nerve injury. BDNF gene therapy can improve RGC survival in experimental models of glaucoma, the leading cause of irreversible blindness worldwide. However, the therapeutic efficacy of BDNF supplementation alone is time limited at least in part due to BDNF receptor downregulation. Tropomyosin-related receptor kinase-B (TrkB) downregulation has been reported in many neurological diseases including glaucoma, potentially limiting the effect of sustained or repeated BDNF delivery.

Here, we characterize a novel adeno-associated virus (AAV) gene therapy (AAV2 TrkB-2A-mBDNF) that not only increases BDNF production but also improves long-term neuroprotective signaling by increasing expression of the BDNF receptor (TrkB) within the inner retina. This approach leads to significant and sustained elevation of survival signaling pathways ERK and AKT within RGCs over 6 months and avoids the receptor downregulation which we observe with treatment with AAV2 BDNF alone. We validate the neuroprotective efficacy of AAV2 TrkB-2A-mBDNF in a mouse model of optic nerve injury, where it outperforms conventional AAV2 BDNF or AAV2 TrkB therapy, before showing powerful proof of concept neuroprotection of RGCs and axons in a rat model of chronic intraocular pressure (IOP) elevation. We also show that there are no adverse effects of the vector on retinal structure or function as assessed by histology and electroretinography in young or aged animals. Further studies are underway to explore the potential of this vector as a candidate for progression into clinical studies to protect RGCs in patients with glaucoma and progressive visual loss despite conventional IOP-lowering treatment.

## Introduction

The eye is at the forefront of the application of gene therapy techniques. This year, LUXTURNA^™^ (voretigene neparvovec, Spark Therapeutics Inc.), a gene therapy to treat the rare inherited retinal disease Leber’s Congenital Amaurosis, became one of the few select adeno-associated virus (AAV) vectors to be approved by the US Food and Drug Administration (www.fda.gov). Although other gene therapies for single gene defect diseases of the retina and optic nerve are progressing through clinical trials (clinicaltrials.gov), gene therapies for diseases like glaucoma, which do not arise from a single gene defect, are lacking.

Glaucoma is the leading cause of irreversible blindness worldwide and is characterized by progressive, permanent damage to the optic nerve resulting in the loss of retinal ganglion cells (RGCs). RGC death results in vision loss, often significantly impacting the quality of life^1^. Reduction in intraocular pressure (IOP) is the most important modifiable risk factor for glaucoma, and all current pharmacological or surgical treatments are designed to reduce IOP, regardless of glaucoma subtype or disease stage^2–4^. However, although this is an effective approach for many glaucoma patients, a proportion continue to deteriorate with approximately 1 in 8 progressing to blindness in at least one eye^5–7^. Therapies designed to target and protect RGCs as an adjunct to IOP lowering are therefore still needed.

We chose to develop and test a novel gene therapy construct involving brain-derived neurotrophic factor (BDNF) and its cognate receptor, tropomyosin-related receptor kinase-B (TrkB)^8–10^, whose expression and activation are impaired in rodent^11–16^, dog^17^, and primate^18^ models of glaucoma. There is also increasing evidence of reduced BDNF and TrkB signaling in human glaucoma^19–22^, including patients with normal tension glaucoma^19^.

Numerous studies have demonstrated that intravitreal delivery of BDNF by injection of recombinant protein^23,24^ or through gene therapy approaches^25–27^ can alleviate RGC loss after optic nerve injury. However, the therapeutic efficacy of BDNF supplementation alone is time limited at least in part due to BDNF receptor downregulation^28–32^, potentially limiting the effect of sustained or repeated BDNF delivery.

Gene therapy approaches using BDNF may also be hampered long-term by the accumulation of unprocessed proBDNF, which needs to be proteolytically cleaved intracellular or extracellularly to generate mature BDNF (mBDNF). Whereas mBDNF preferentially binds the TrkB receptor and is associated with neuronal survival, proBDNF binds p75^NTR^ receptors associated with apoptosis^33–35^. Inefficiencies to process large levels of proBDNF may compromise the benefits of using AAV BDNF^36^.

To overcome BDNF receptor downregulation and enhance neuroprotective BDNF signaling on a timescale relevant to chronic neurodegenerative disease, we have developed a novel gene therapy construct containing coding sequences for both TrkB and mBDNF separated by a viral-2A peptide linker^37^. This gene construct, controlled by the constitutive cytomegalovirus enhancer/chicken beta-actin (CAG) promoter, generates a single transgene which is subsequently processed intracellularly through cutting within the viral-2A site to produce two mature proteins. These two separate proteins are then directed to their respective intracellular sites with the TrkB receptors targeted to the cell surface and the mBDNF targeted to secretory vesicles from which the protein may be released extracellularly^37^. We have shown that this gene therapy construct elevates both mature BDNF and TrkB in vitro and in vivo and can provide effective neuroprotection to SH-SY5Y cells against oxidative damage^37^. The aim of the current study was to measure the capacity of this gene therapy to protect RGC in in vivo models of optic nerve crush (ONC) and IOP elevation. We also explored the mechanisms behind the RGC neuroprotection mediated by the vector and the long-term structural and functional effects of chronic transgene expression in adult and aged animals.

## Materials and methods

### Vector production

AAV2 vectors were produced by Vigene Biosciences (9430 Key West Avenue, Suite 105, Rockville, MD 20850, USA). Vector particles were liberated following freeze–thaw of HEK293 cells transfected with plasmid DNA, followed by iodixanol gradient ultracentrifugation, de-salting and were suspended in phosphate-buffered saline (PBS) buffer (Thermo Fisher Scientific). Titres were confirmed by Vigene Biosciences through qPCR using primers recognizing the ITR regions. All vectors (unless stated otherwise) contained a modified CAG promoter, shortened Woodchuck Hepatitis Virus Posttranscriptional Regulatory Element (WPRE), and simian virus 40 late polyadenylation signal (polyA), as described previously^37^ (Supplementary Figure 1a).

### Intravitreal vector injection

**Fig. 1:**
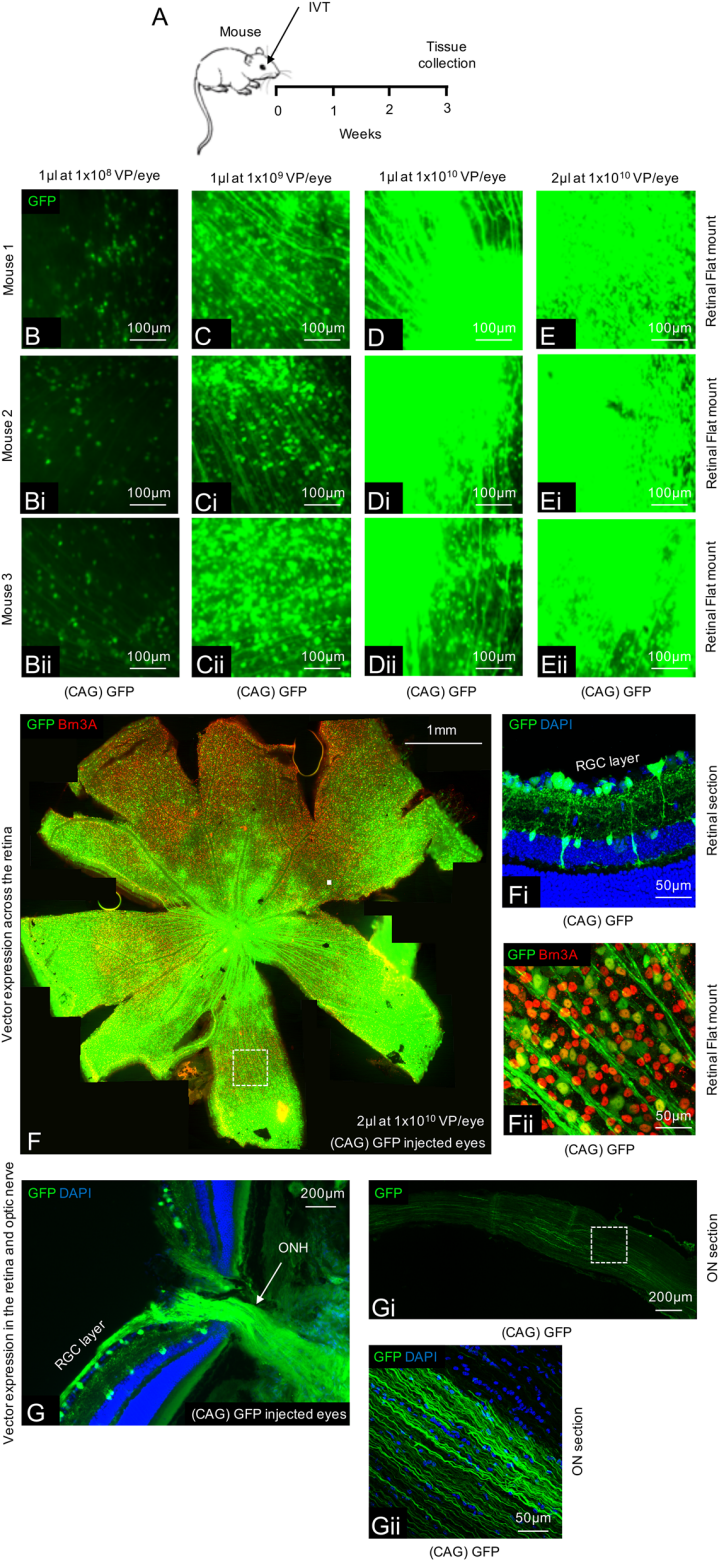
Ensuring adequate transfection in the mouse retina using an AAV2 CAG vector system. **a** Schematic of the procedure performed. **b**–**e** Expression of GFP throughout the retina using various titres and volumes of AAV2 GFP (*n* = 4/group). **f** A tile scan of a representative retinal wholemount 3 weeks after injection of 2 µl AAV2 GFP at 1 × 10^10^ vector particles/eye. **f**i GFP expression is localized to the inner retina with transduction of predominantly RGCs and some amacrine/Müller glia cells. **f**ii Magnification of the wholemount shows co-localization between GFP and Brn3A+ RGCs. **g**–**g**ii GFP expression can also be seen transported anterogradely down the optic nerve in both proximal (**g**) and distal (**g**i, ii) locations

Adult, male C57BL/6 mice (Charles River Laboratories) or Sprague Dawley rats (Charles River Laboratories) were anesthetized with intraperitoneal injection of ketamine (50 mg/kg) and xylazine (10 mg/kg) and given topical 1% tetracaine (Bausch & Lomb) eye drops prior to injection. Freshly thawed AAV2 vectors were diluted to working stock in sterile PBS and 2 μl (mice) or 5 μl (rats) of vector was injected through the sclera into the vitreous of the eye approximately 3 mm posterior to the superior-temporal limbus (Syringe: 5 μl, #65RN; Needle: ga33, 8 mm, pst2, Hamilton Co.). Care was taken to avoid penetration of the lens or damage to the vortex veins during the intravitreal injection. Injections were given slowly over 20 s to allow diffusion of the vector suspension and to allow IOP equilibration before needle removal. All injections were carried out by the same surgeon. Injection of vectors at lower titres of 1 × 10^8^ and 1 × 10^9^ Viral particles (VP)/eye transfected a limited number of RGCs and expression within those cells was relatively low (Fig. 1a–cii). Injection of vector at 1 × 10^10^ VP/eye provided optimal vector spread and transfection of the majority of RGCs (Fig. 1d–fii). Therefore 1 × 10^10^ VP/eye was used for all experiments, unless stated otherwise. Animals were culled at the indicated time points via CO_2_ inhalation or further procedures were carried out as listed below.

### Experimental design

All in vivo work was carried out in accordance with the UK Home Office regulations for the care and use of laboratory animals, the UK Animals (Scientific Procedures) Act (1986), and the Association for Research in Vision and Ophthalmology’s Statement for the Use of Animals in Ophthalmic and Visual Research. Cohorts of animals processed for this project were as follows: Cohort 1: AAV2 GFP titre comparison study; *n* = 16 mice. Cohort 2: AAV2 TrkB-2A-mBDNF expression in the retina and optic nerve study; *n* = 12 mice. Cohort 3: AAV2 TrkB-2A-mBDNF long-term expression and signaling study; *n* = 36 mice. Cohort 4: AAV2 BDNF long-term study; *n* = 25 mice. Cohort 5: AAV2 TrkB-2A-mBDNF safety and functional testing study; *n* = 22 mice. Cohort 6: AAV2 TrkB-2A-mBDNF vs AAV2 BDNF optic nerve crush neuroprotection study; *n* = 30 mice. Cohort 7: AAV2 TrkB-2A-mBDNF vs AAV2 TrkB optic nerve crush neuroprotection and function study; *n* = 40 mice. Cohort 8: AAV2 TrkB-2A-mBDNF laser-induced ocular hypertension study; *n* = 30 rats.

### Mouse optic nerve crush

Twenty-one days after intravitreal vector injection, mice underwent an ONC procedure. Under a binocular operating scope, a small incision was made with spring scissors in the conjunctiva beginning inferior to the globe and around the eye temporally. This exposed the posterior aspect of the globe, allowing visualization of the optic nerve. The exposed optic nerve was grasped approximately 1–3 mm from the globe with cross-action forceps (Dumont #N7 cat. #RS-5027; Roboz) for 10 s. After 10 s the optic nerve was released, the forceps removed, and the eye rotated back into place. Contralateral fellow eyes served as untreated controls. Seven or 10 days after ONC, mice were culled via CO_2_ inhalation.

### Trabecular laser-induced IOP elevation in rat

Twenty-one days after intravitreal vector injection, ocular hypertension was induced in rats using a modification of the method developed by Levkovitch-Verbin et al.^38^, and fully described previously^39^. Rats were placed in front of a slit-lamp and 40–60 laser pulses (wavelength 532 nm, spot size 50 μm, power 700 mW, duration 600 ms) were directed around the circumference of the trabecular meshwork to impair aqueous drainage. Rats underwent two laser procedures, 1 week apart, as the elevation in IOP is refractory. Contralateral fellow eyes served as untreated controls. IOP was measured bilaterally under anesthesia on procedure days and whilst awake at subsequent time points using a TonoLab rebound tonometer (Tiolat Oy). Tonometry was performed within 5 min of anesthesia onset and between the hours of 9:00 a.m. and 11:00 a.m. Fifteen recordings were taken from each eye at each time point. Forty-two days after the first laser procedure, rats were perfused transcardially with 4% paraformaldehyde (PFA) under terminal anesthesia and eyes and nerves collected.

### Electroretinography

Twenty-one days after intravitreal vector injection and 3 and 7 days after ONC, full-field ERGs were recorded simultaneously from both eyes of mice, as described previously^40^. Animals were dark-adapted overnight (>12 h) and ERG recordings took place in the dark under low level, red light illumination. Pupil dilation was achieved using 0.5% tropicamide (Bausch & Lomb) and 2.5% phenylephrine (Bausch & Lomb). ERG recordings were acquired using an Espion E3 system with full-field Ganzfeld sphere (Diagnosys, Cambridge, UK) as follows: Scotopic threshold response recorded at −4.73 log cd.s.m^−2^ (averaged responses from between 20 recordings with interstimulus interval (ISI) of between 3 s). Peak amplitude between 80 and 120 ms was taken as positive scotopic threshold response (pSTR) amplitude, indicating RGC activity. The B-wave was recorded at −1.90 log cd.s.m^−2^ averaging 2 recordings with 20 s ISI. Peak amplitude between 70 and 110 ms was taken as B-wave amplitude. B-wave amplitude indicates bipolar cell and Müller cell activity. Mixed A-wave/B-wave recording were measured at 1.29 log cd.s.m^−2^ from a single flash. A-wave amplitude was taken as trough amplitude within the first 20 ms and represents photoreceptor activity. ERGs were performed in both young (28 days) and older (360 days) mice. Aged animals were used as most glaucoma patients are older and aged animals might be expected to be more vulnerable to adverse effects of treatment.

### Western blots

Retinas were excised from the eye cup immediately after death and tissue snap frozen on dry ice prior to lysing using a Lysis-M reagent containing cOmplete Mini Protease Inhibitor (Roche) and phosphatase inhibitors (Thermo Fisher Scientific). Following 20 min homogenization, tissue was centrifuged at 13,000 rpm for 10 min to isolate the soluble cell extract. Protein concentration was determined using a bicinchoninic acid (BCA) protein assay (Thermo Fisher Scientific) and equal quantities of protein loaded into 10% or 4–12% Bis–Tris gels (NuPAGE Novex, Thermo Fisher Scientific). Membranes were blocked in 5% dried skimmed milk in PBS with 0.2% Tween20 (Sigma-Aldrich) for 60 min and then incubated overnight at 4 °C in primary antibody (Supplementary Table 1A). Primary antibodies were visualized with HRP conjugated anti-rabbit secondary antibody (Vector Laboratories; 1:8000) and signal detection using ECL Prime (GE Healthcare) and an Alliance Western blot imaging system (UVItec Ltd.).

### Immunohistochemistry

#### Sections

Eyes were fixed by placing the globe in 4% PFA overnight followed by dehydration in 30% sucrose in PBS at 4 °C (>24 h) and embedding in silicon molds containing optimal cutting temperature compound (OCT) (Sakura Finetek). Eyes were then frozen on dry ice and sectioned at 13 μm through the dorsal–ventral/superior–inferior axis of the retina onto superfrost plus slides (VWR), using a Bright OTF 5000 cryostat (Bright Instruments). Sections were simultaneously blocked and permeabilized by incubation in 5% normal goat serum (NGS) in PBS with 0.3% Triton X-100 and 2% bovine serum albumin (BSA) for 60 min at room temperature. Sections were then incubated in primary antibody (Supplementary Table 1A) diluted in blocking solution overnight at 4 °C, before incubation with secondary antibody (Supplementary Table 1B) diluted in blocking solution for 120 min at room temperature. Nuclei were counterstained with DAPI. Sections were imaged using a Leica DM6000 epifluorescence microscope (Leica Microsystems) and high magnification images achieved using a SP5 confocal microscopy equipped with a 40× lens (Leica Microsystems). Fluorescence within individual RGCs was calculated as corrected total cell fluorescence (CTCF) using an established quantification protocol^41^. Using the freehand tool in ImageJ, individual RGCs were outlined according to positive TUJ1 staining. The fluorescent channel was then switched to either p-TrkB, p-ERK, or p-AKT and the integrated density measurement taken. A background for each image was also obtained adjacent to the RGC layer. The following calculation was applied to 30–71 RGCs per time point: CTCF = integrated density − (area of selected cell × mean fluorescence of background reading) and individual data points plotted.

#### Flatmounts

Retinal flatmounts were prepared following dissection of the posterior eye structure and removal of the lens. The retinas were gently dissociated from the underlying retinal pigment epithelium, flattened and post-fixed for 30 min in 4% PFA prior to staining. Retinal flatmounts were washed in 0.5% Triton X-100 in PBS and frozen at −80 °C for 10 min to permeate the nuclear membrane and improve antibody permeation before blocking in 10% normal donkey serum (NDS), 2% BSA, and 2% Triton X-100 in PBS for 60 min. RGCs were labeled with Brn3A (Supplementary Table 1A) and imaged using a 20× objective. RGC counts were measured by a third party concealed to the treatments. Individual, RGC counts were performed using ImageJ and an image-based tool for counting nuclei (ITCN) plugin. Eight images were used to quantify RGCs across the mouse retina and eight central and sixteen peripheral images to assess RGC survival in rats. RGC density was expressed as RGCs/mm^2^.

#### Optic nerve sections

A selection of optic nerves were also visualized for expression of TrkB and BDNF with nerves sectioned longitudinally (13 μm) and labeled and imaged as stated above (“Sections” and Supplementary Table 1A and B).

### Cross-sectional assessment of axons in the optic nerve

Optic nerves were cut immediately posterior to the globe and carefully removed from the skull and brain to allow dissection of the entire length of the optic nerve from the optic chiasm. The optic nerves were then immersion fixed in 5% glutaraldehyde, washed in PBS, and post-fixed using 1% osmium tetroxide for 180 min. Following the fixation step, the nerves were washed, dehydrated through an ascending alcohol series (70% for 15 min; 95% for 15 min; 100% for 3 × 15 min), and immersed in an ascending propylene oxide series with resin for 2 days as previously described^39^. On the final day, diluted resin was replaced by 100% resin and the optic nerves were placed in molds and heated to 65 °C for 48 h. 1 μm thick optic nerve sections were collected on VWR poly-lysine slides using a Leica EM UC6 ultra-microtome (Leica Microsystems). Once dried and adhered to the slide, semi-thin sections were etched to remove the resin around the nerves (30 s in a solution of 10 mL propylene oxide, 10 mL 100% ethanol, 10 pellets NaOH) and stained for 30 s using a Richardson stain (1% methylene blue, 1% borax (sodium borate), 1% Azure II) prior to washing with distilled water. The slides were cover-slipped with DPX mounting medium prior to imaging.

The entire cross-sectional area of the nerve was imaged using a bright field microscope with Lucia G software and a 10× objective. A 100× oil immersion objective was then used to capture three high-resolution representative images of each optic nerve. Axons within these images were blind counted using Image-J and an established plugin^39^ and numbers extrapolated to estimate the entire number of axons remaining after injury.

### Statistical analysis

Data is shown as mean ± SEM or, where stated, mean ± SD for all survival-based studies involving mice and rats. Data was analyzed using Student’s two-sample *t*-tests or when comparing between three or more groups, a one-way ANOVA followed by Bonferroni-modified *t*-tests for multiple comparisons if *P* < 0.05.

## Results

**Fig. 2:**
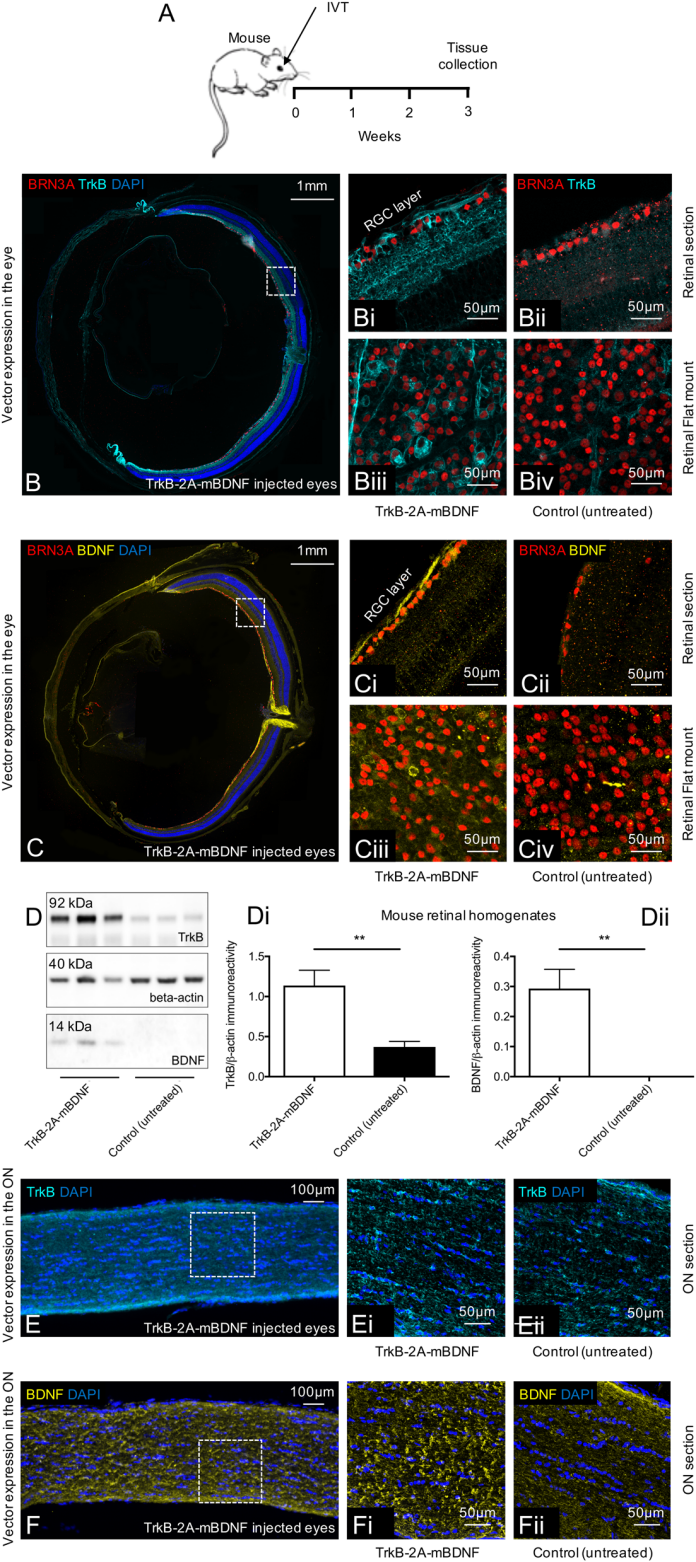
Expression of TrkB and BDNF in mouse eyes 3 weeks after intravitreal delivery of AAV2 TrkB-2A-mBDNF (2 µl, 9.14 × 10^9^ vector particles/eye). **a** Schematic of the procedure performed. **b**–**b**iv Increased TrkB receptor expression on the cell surface of transduced RGCs (Brn3A+ cells) throughout the mouse retina after treatment with AAV2 TrkB-2A-mBDNF compared to untreated eyes. **c**–**c**iv Increased BDNF expression within transduced RGCs compared to untreated eyes (*n* = 3 for retinal sections, *n* = 3 for retinal flat mount). **d**–**d**ii Increased TrkB and BDNF expression was detected in retinal lysates following AAV2 TrkB-2A-mBDNF delivery compared to contralateral, untreated eyes (*n* = 6), **P < 0.01. **e**–**e**ii TrkB receptor expression did not appear elevated within the optic nerve 3 weeks after AAV2 TrkB-2A-mBDNF delivery (*n* = 4). **f**–**f**ii Increased BDNF anterograde transport was observed in the optic nerve proximal and distal to the globe after intravitreal injection of AAV2 TrkB-2A-mBDNF (*n* = 4). Graphs show mean ± SEM with *P* values obtained via Student’s two-sample *t*-tests

Processing and expression of the single transgene coding for both TrkB and mBDNF was examined in mouse eyes and optic nerves following intravitreal injection of AAV2 TrkB-2A-mBDNF (Fig. 2). Three weeks after injection of the vector, high levels of TrkB and BDNF expression were observed in the RGC layer of the retina (Fig. 2b, c). Western blots of mouse retinal homogenates showed that TrkB receptor immunoreactivity was increased 3-fold in mice treated with TrkB-2A-mBDNF compared to untreated control animals (*P* < 0.01; Fig. 2d, di). Western blotting also confirmed that there was efficient processing of the single transgene (TrkB receptors 80–140 kDa and mBDNF 14 kDa), supporting previous observations^37^ that the novel construct was cleaved correctly in vivo. New TrkB receptors were expressed on the RGC membrane (Fig. 2bi, iii) whilst BDNF was seen within RGCs (Fig. 2ci, iii), an observation below detection in controls imaged at the same intensity (Fig. 2cii, iv). Intravitreal injection of AAV2 TrkB-2A-mBDNF also led to enhanced BDNF transportation along axons of the optic nerve (Fig. 2f, fi), as also seen with anterograde transport of GFP following intravitreal injection of AAV2 GFP (Fig. 1g–gii), whilst the TrkB receptor appeared localized to the retina (Fig. 2e, ei).

**Fig. 3:**
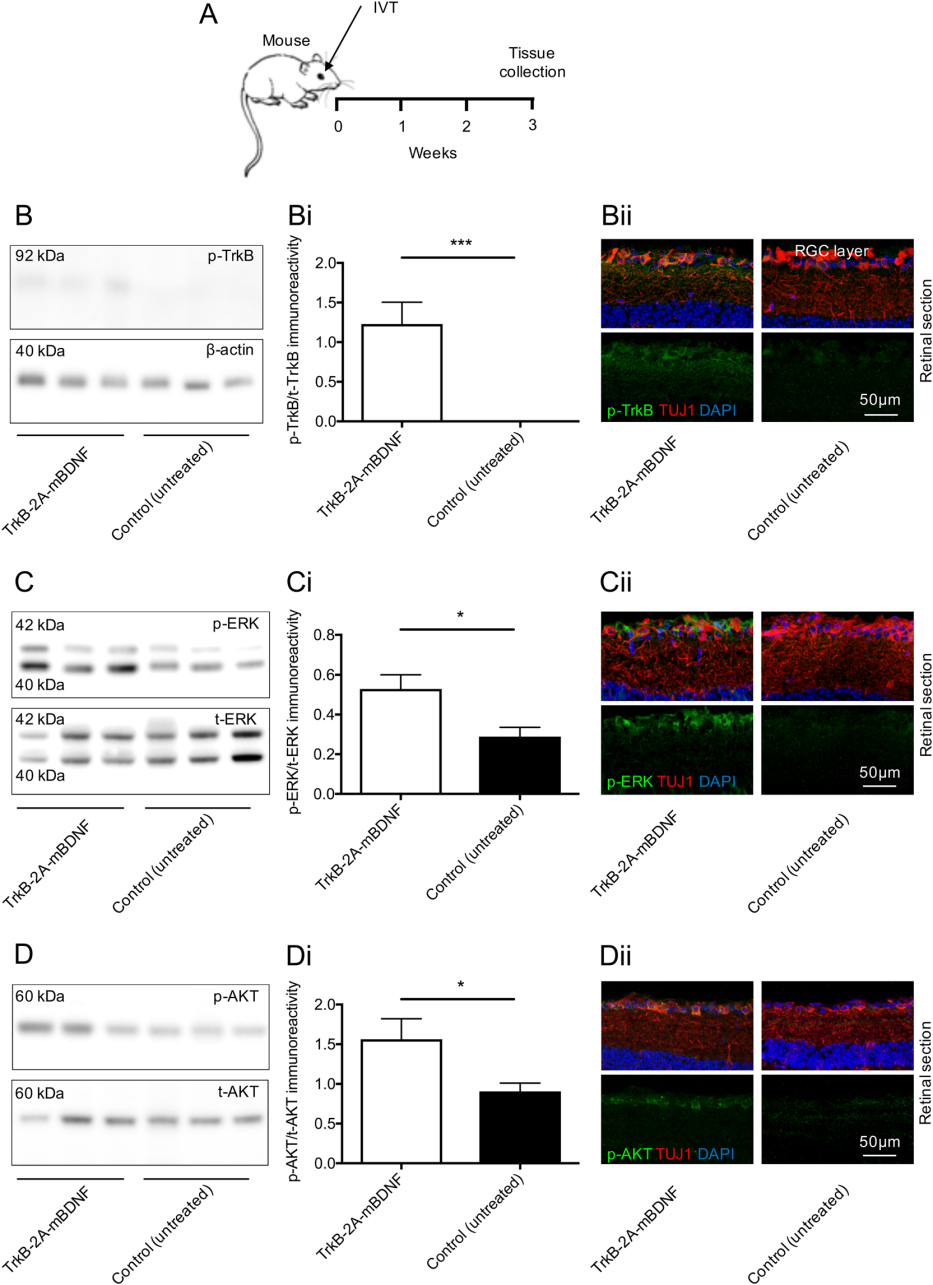
Elevated BDNF/TrkB signaling was measurable in the mouse retina 3 weeks after intravitreal injection of AAV2 TrkB-2A-mBDNF (2 µl, 9.14 × 10^9^ vector particles/eye). **a** Schematic of the procedure performed. **b**, **b**i Increased phosphorylated TrkB(Y515) was detected in retinal lysates following AAV2 TrkB-2A-mBDNF delivery compared to contralateral, untreated eyes (*n* = 6). **b**ii p-TrkB immunoreactivity was upregulated specifically in RGCs (TUJ1+ cells) after treatment with AAV2 TrkB-2A-mBDNF (*n* = 3). **c**, **c**i Increased phosphorylated ERK was detected in retinal lysates following AAV2 TrkB-2A-mBDNF delivery compared to contralateral, untreated eyes (*n* = 6). **c**ii p-ERK immunoreactivity was upregulated in RGCs (TUJ1+ cells) after treatment with TrkB-2A-mBDNF compared to untreated eyes (*n* = 3). **d**, **d**i Phosphorylated AKT was upregulated in retinas transduced with AAV2 TrkB-2A-mBDNF compared to untreated eyes (*n* = 5). **d**ii p-AKT immunoreactivity was upregulated in RGCs (TUJ1+ cells) after treatment with AAV2 TrkB-2A-mBDNF compared to untreated eyes (*n* = 3). *P < 0.05 and ***P < 0.001. Graphs show mean ± SEM with *P* values obtained via Student’s two-sample *t*-tests

Western blotting and immunohistochemistry after intravitreal injection of AAV2 TrkB-2A-mBDNF also showed a marked up-regulation in TrkB receptor signaling, as shown by the significant increase in phosphorylated TrkB expression (p-^Y515^TrkB, *P* < 0.05; Fig. 3b, bi) and in the ratio of phosphorylated/total ERK (p-ERK; AAV2 TrkB-2A-mBDNF = 0.53 ± 0.07, control (untreated) = 0.29 ± 0.05, *P* < 0.05; Fig. 3c, ci) and AKT expression (p-AKT; AAV2 TrkB-2A-mBDNF = 1.56 ± 0.26, control (untreated) = 0.91 ± 0.10, *P* < 0.05; Fig. 3d, di) compared to untreated control eyes. Immunohistochemistry using antibodies directed at the active, phosphorylated TrkB receptor (p-TrkB) and downstream signaling pathway proteins ERK and AKT demonstrated an increase in staining exclusively in RGC layer cells (see Fig. 3bii, cii, dii).

**Fig. 4:**
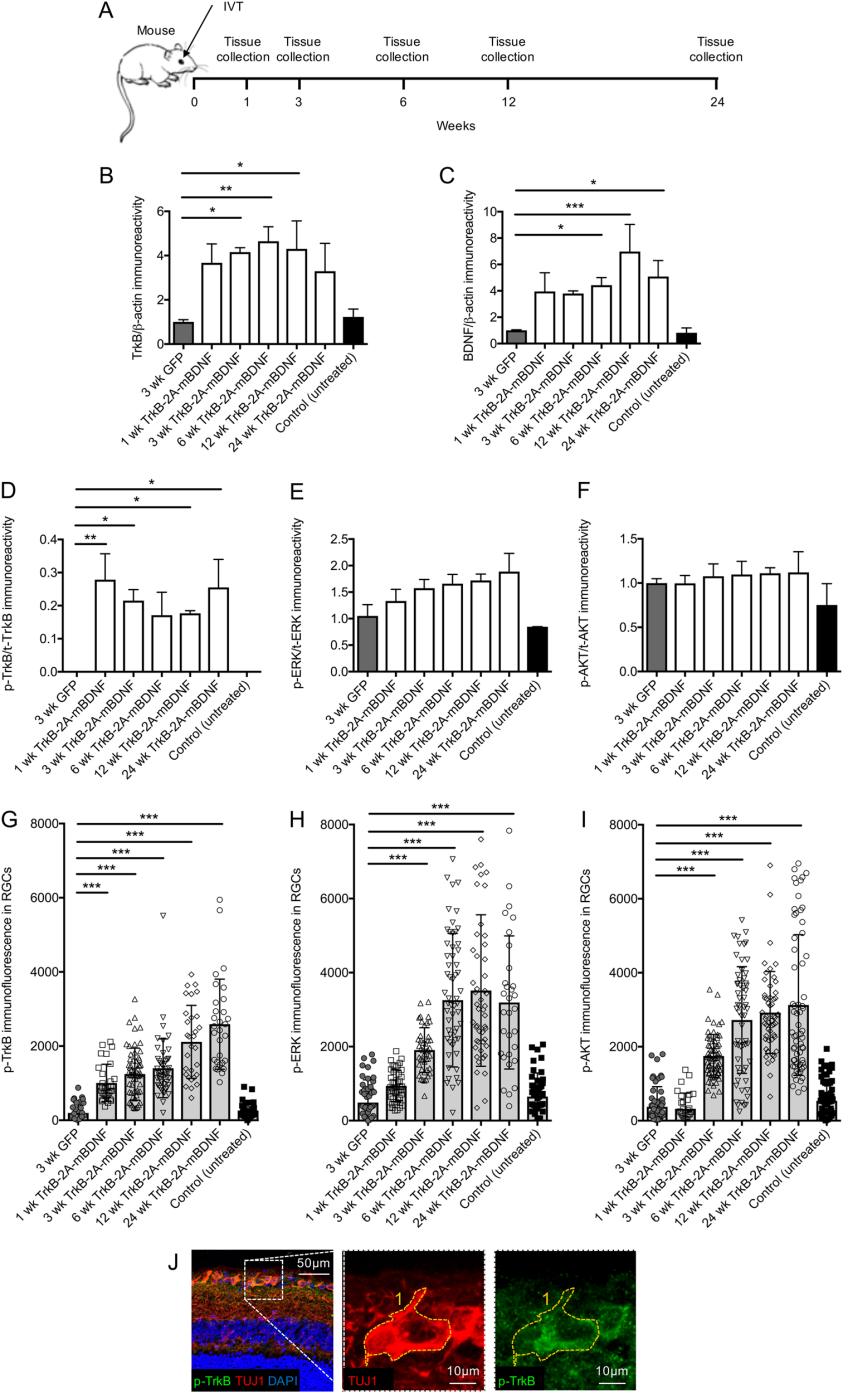
Long-term vector expression and signaling in the mouse retina after intravitreal injection of AAV2 GFP or AAV2 TrkB-2A-mBDNF (2 µl, 1 × 10^10^ vector particles/eye). **a** Schematic of the procedure performed and the time points in which tissues were collected. **b**, **c** Increased TrkB and BDNF expression was detected in retinal lysates at multiple time points post injection of AAV2 TrkB-2A-mBDNF compared to AAV2 GFP (*n* = 3/time point). **d** Increased p-TrkB was measured in retinal lysates expressing AAV2 TrkB-2A-mBDNF compared to AAV2 GFP or untreated controls (*n* = 3/time point). **e**, **f** Activated ERK (p-ERK) and AKT (p-AKT) showed a trend for increased expression following transduction with AAV2 TrkB-2A-mBDNF when assessed in whole retinal lysates (*n* = 3/time point). **g**–**i** p-TrkB, p-ERK, and p-AKT immunofluorescence within individual RGCs (TUJ1+ cells) was significantly increased in eyes injected with AAV2 TrkB-2A-mBDNF compared to controls (*n* = 2000 RGCs from 14 retinas). **j** Representative image showing how immunofluorescence was measured within individual RGCs (TUJ1+ cells). *P < 0.05, **P < 0.01 and ***P < 0.001 compared to 3-week GFP. Graphs show mean ± SEM with *P* values obtained via a one-way ANOVA followed by Bonferroni-modified *t*-tests for multiple comparisons

The density of TrkB and BDNF transgene expression was also measured at regular time points over 6 months following a single intravitreal injection of AAV2 TrkB-2A-mBDNF (Fig. 4a). Over 24 weeks, TrkB expression remained upregulated in the AAV2 TrkB-2A-mBDNF group having an average 3–4-fold greater expression of TrkB compared to levels in control, untreated mice and mice injected with a control AAV2 CAG-GFP vector (TrkB; AAV2 TrkB-2A-mBDNF = 4.16 ± 0.20, AAV2 GFP = 1.00 ± 0.10 at 3 weeks, *P* < 0.05; Fig. 4b). Increased BDNF transgene expression was seen 1 week after AAV2 TrkB-2A-mBDNF injection and was maintained to week 24 (BDNF; AAV2 TrkB-2A-mBDNF = 5.08 ± 1.21, AAV2 GFP = 1.00 ± 0.04, control (untreated) = 0.82 ± 0.30 at 24 weeks, *P* < 0.01; Fig. 4c). Phosphorylated TrkB expression was also shown to be elevated in the AAV2 TrkB-2A-mBDNF group compared to controls indicating enhanced kinase activity and signaling (Fig. 4d). Phosphorylated ERK and AKT levels in retinal homogenates showed a similar trend in elevated activity but likely due to the diluting effect of using the entire retinal homogenate (RGCs represent 1% of the entire retinal homogenate) it was not possible to detect significant differences between groups (*P* > 0.05; Fig. 4e, f).

However, quantification of p^Y515^-TrkB, p-ERK, or p-AKT fluorescence in specific RGCs (TUJ1+ cells, as shown in Fig. 4j) demonstrated a marked increase in activity after treatment with AAV2 TrkB-2A-mBDNF compared to controls which remained at basal levels (Fig. 4g–i). Quantitative immunocytochemical analysis of p^Y515^-TrkB immunoreactivity within RGCs showed a significant and selective up-regulation at all time points (p-TrkB; AAV2 TrkB-2A-mBDNF = 1245 ± 90.4, AAV2 GFP = 198.3 ± 50.9 at 3 weeks, *P* < 0.001; Fig. 4g). Likewise, immunofluorescence of p-ERK and p-AKT were significantly elevated from week 3 after AAV2 TrkB-2A-mBDNF injection and by 6 weeks was 6-fold higher than levels seen in either control without evidence of attenuation at later time points (p-ERK; AAV2 TrkB-2A-mBDNF = 3255 ± 217, AAV2 GFP = 487.8 ± 89.6; p-AKT; AAV2 TrkB-2A-mBDNF = 2721 ± 185, AAV2 GFP = 374.5 ± 75.7 at 6 weeks, *P* < 0.001; Fig. 4h, i).

In contrast, expressing BDNF alone using an AAV2 BDNF construct (Supplementary Figure 2) led to a rapid downregulation of TrkB receptors in the retina (Supplementary Figure 2b). TrkB expression decreased at 1 week after AAV2 BDNF intravitreal injection and continued to decline over time (TrkB; AAV2 BDNF = 0.40 ± 0.02, AAV2 GFP = 0.89 ± 0.11-fold at 1 week relative to control (untreated) eyes, *P* < 0.001, Supplementary Figure 2b). By week 20, TrkB expression in the retina after treatment with AAV2 BDNF was 4-fold lower than control levels (TrkB; AAV2 BDNF = 0.23 ± 0.02, AAV2 GFP = 0.84 ± 0.06-fold relative to control (untreated) eyes, *P* < 0.01 Supplementary Figure 2c). AAV2 TrkB-2A-mBDNF stably increased TrkB expression compared to naive and AAV2 GFP injected eyes with a 2–3-fold increase in receptor expression (TrkB; AAV2 BDNF = 2.39 ± 0.33-fold relative to control (untreated) eyes, Supplementary Figure 2c). Immunohistochemical analysis confirmed a downregulation of TrkB expression on the surface of RGCs in the AAV2 BDNF group (Supplementary Figure 2d–f) and analysis of p^Y515^-TrkB immunoreactivity indicated a weaker survival signaling response compared to AAV2 TrkB-2A-mBDNF at 5 months (Supplementary Figure 2h). Although no adverse effects of using AAV2 BDNF were measured over time (Supplementary Figure diii–fiv and Supplementary Table 2A), increased levels of uncleaved, proBDNF within the retina were detected (AAV2 BDNF = 558.18 ± 51.3, control (untreated) = undetectable, Supplementary Figure 2g) which could pose a damaging effect during disease progression, when p75 expression increases^42^. ProBDNF expression was not detected in AAV2 GFP or AAV2 TrkB-2A-mBDNF transduced eyes (Supplementary Figure 2h).

**Fig. 5:**
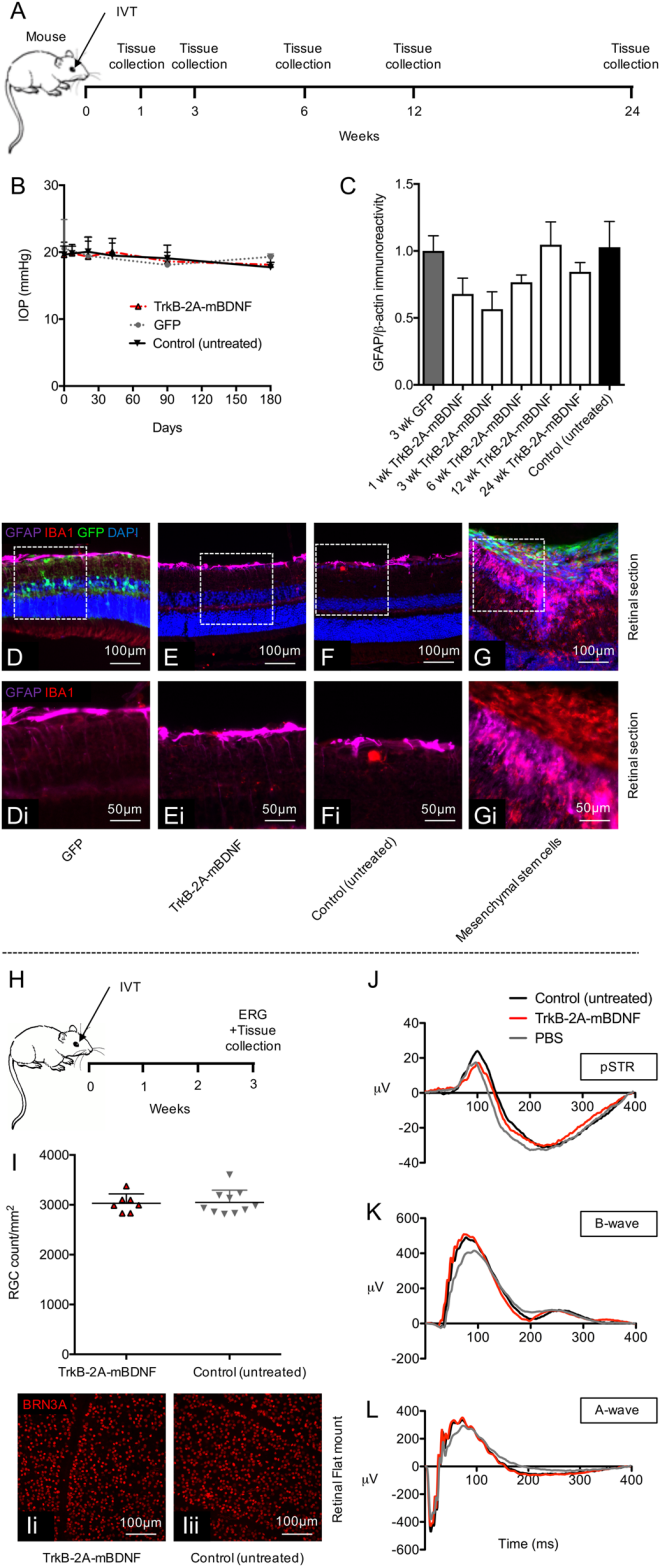
Delivery of AAV2 TrkB-2A-mBDNF (2 µl, 1 × 10^10^ vector particles/eye) to the mouse retina had no adverse effects on retinal health or function. **a** Schematic of the procedure performed and the time points in which tissues were collected. **b** Intraocular pressure (IOP) did not change pre- and post-injection with AAV2 GFP or AAV2 TrkB-2A-mBDNF compared to control, non-injected eyes (*n* = 5/time point). **c** Gliosis, measured by GFAP expression over time from AAV2 treated retinal lysates compared to control, non-injected eyes (*n* = 3/time point) also showed no increase in reactivity. **d**–**f** Representative images of GFAP immunoreactivity and inflammatory cell marker IBA1 in retinal sections 3 weeks post-treatment. **g** Gliosis and substantial inflammation was observed 3 weeks after intravitreal injection of 20,000 GFP+ mesenchymal stem cells (2 µl) into the eye, serving as a positive control. **i**–**i**ii RGC (Brn3A+ cell) counts 3 weeks after intravitreal injection of AAV2 TrkB-2A-mBDNF compared to untreated control eyes showed no detrimental effect on cell number (*n* = 7–10). **j**–**l** Representative pSTRs, characteristic of RGC function, A-wave (bipolar and Müller activity), and mixed A-wave/B-wave (photoreceptor function) profiles between untreated, vector treated, or PBS injected eyes were also not altered when transduced with AAV2 TrkB-2A-mBDNF (*n* = 7–8) **h** Schematic of the procedure performed and the time points in which tissues were collected and ERGs recorded

Assessment of anatomical changes to the retinal layers was also measured over 6 months after treatment with AAV2 TrkB-2A-mBDNF (Fig. 5). Immunohistochemical staining with antibodies against glial-fibrillary acidic protein (GFAP) did not reveal any evidence for gliosis (*P* > 0.05; Fig. 5c–fi), nor was there evidence of significant microglia activation, as shown by IBA1 staining (Fig. 5d–fi) which contrasts with the effects of other potentially neuroprotective treatments we have explored such as the use of mesenchymal stem cells (Fig. 5g, gi)^43^. We also found no significant adverse response to AAV2 injection and expression of transgenes (TrkB and mBDNF) as assessed by measures of retinal morphology (Fig. 5d–fi), RGC number (Fig. 5i–iii) and IOP (Fig. 5b).

Electroretinography performed on mice intravitreally injected unilaterally with AAV2 TrkB-2A-mBDNF also revealed no adverse effects of vector administration on visual function (Fig. 5j–l). pSTR responses were not statistically different between control, untreated eyes = 23.1 ± 2.7 μV, PBS-treated eyes = 17.8 ± 2.7 μV, or AAV2 TrkB-2A-mBDNF-treated eyes = 19.6 ± 4.6 μV (*P* = 0.559; Fig. 5j), nor were A-wave recordings (control = −461 ± 52 μV, PBS = −395 ± 42 μV, TrkB-2A-mBDNF = −446 ± 63 μV, *P* = 0.662; Fig. 5l), or B-wave recordings (control = 536 ± 65 μV, PBS = 428 ± 68 μV, TrkB-2A-mBDNF = 566 ± 96 μV, *P* = 0.435; Fig. 5k) in aged (1 year) mice, more representative of glaucoma patients who would receive a gene therapy.

**Fig. 6:**
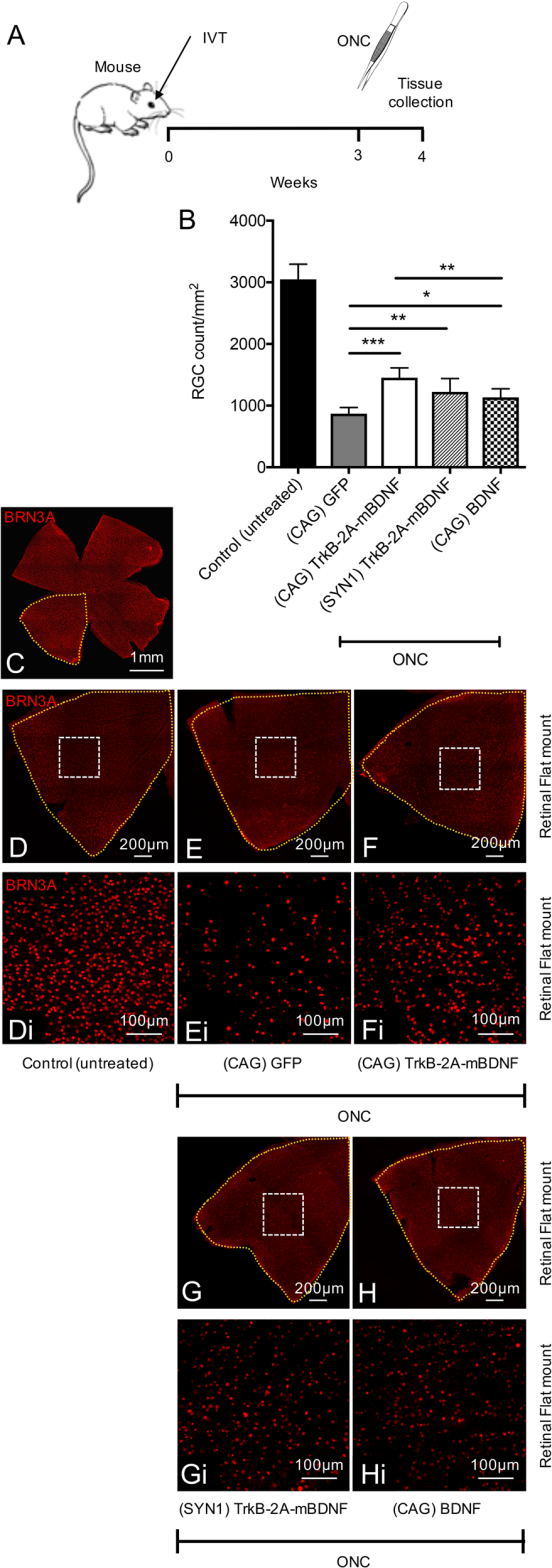
Neuroprotection using AAV2 TrkB-2A-mBDNF vs AAV2 BDNF in a mouse optic nerve crush (ONC) injury model. **a** Schematic of the procedure performed. Vectors were administered 3 weeks prior to ONC (2 µl, 9.14 × 10^9^ vector particles/eye). **b** RGC (Brn3A+ cells) were counted 1 week after ONC, 8 images quantified/retina. **c** Representative image of an entire mouse retinal flat mount. **d**–**h**i Representative image of a retinal quadrant showing the size of a single zoomed in image (white box) which was quantified and used to estimate RGC number. Control (untreated) *n* = 10, (CAG) GFP *n* = 9, (CAG) TrkB-2A-mBDNF *n* = 7, (SYN1) TrkB-2A-mBDNF *n* = 6, (CAG) BDNF *n* = 7. *P < 0.05, **P < 0.01 and ***P < 0.001 compared to AAV2 GFP transduced eyes which had ONC. The graph shows mean ± SD with *P* values obtained via a one-way ANOVA followed by Bonferroni-modified *t*-tests for multiple comparisons. CAG = cytomegalovirus/chicken beta-actin hybrid promoter, SYN1 = synapsin-1 promoter

The neuroprotective capabilities of AAV2 TrkB-2A-mBDNF were then tested in mouse models of ONC to investigate whether increased TrkB and BDNF expression and signaling could protect RGCs from injury (Fig. 6). AAV2 TrkB-2A-mBDNF vectors under the control of CAG or Synapsin-1 (SYN1) promoters were examined, the latter tested to see if more specific neuronal targeting would be advantageous. AAV2 CAG GFP was used as a control vector and AAV2 CAG BDNF as a benchmark for our novel construct, only expressing unmodified BDNF which we have previously shown to be neuroprotective^25^.

Seven days following ONC, eyes injected with AAV2 CAG GFP had lost a significant proportion of RGCs (868 ± 102 RGCs per mm^2^) compared to uncrushed controls (control, 3049 ± 247 RGCs per mm^2^, *P* < 0.001; Fig. 6b–ei). Pre-treatment of mice with either AAV2 CAG TrkB-2A-mBDNF (1454 ± 160 RGCs per mm^2^) or AAV2 SYN1 TrkB-2A-mBDNF (1223 ± 216 RGCs per mm^2^) promoted significant RGC survival after ONC with respect to eyes that received AAV2 CAG GFP (*P* < 0.01; Fig. 6b–hi). Greater level of RGC survival with AAV2 CAG TrkB-2A-mBDNF demonstrated that the CAG promoter was more efficacious in driving transgene expression than synapsin-1 in RGCs.

ONC eyes treated with AAV2 CAG BDNF (1135 ± 139 RGCs per mm^2^) also had a significantly greater number of spared RGCs compared to AAV2 CAG GFP, however, the vector was significantly less effective than AAV2 CAG TrkB-2A-mBDNF (*P* < 0.01; Fig. 6b–hi).

**Fig. 7:**
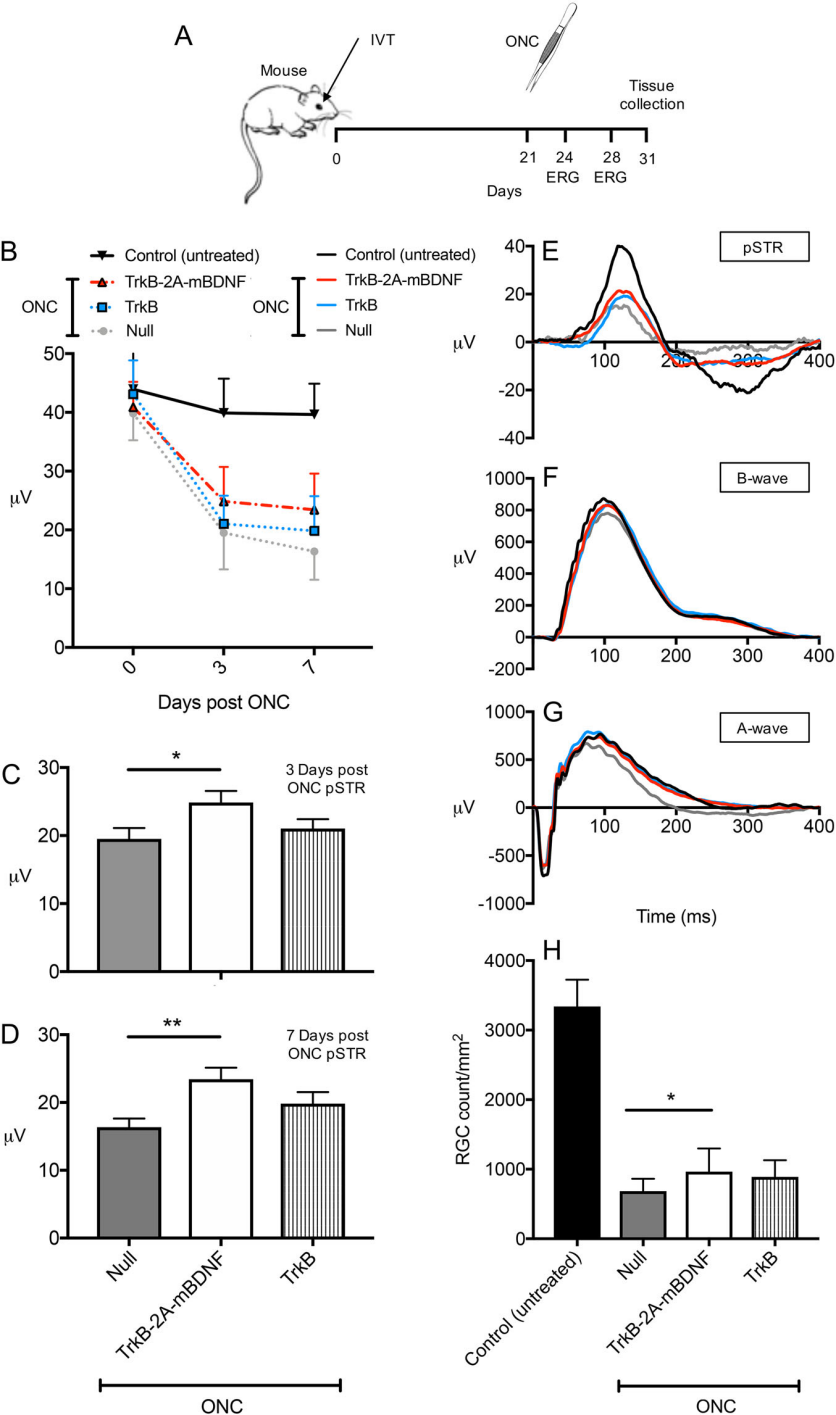
Neuroprotection and functional improvement using AAV2 TrkB-2A-mBDNF vs AAV2 TrkB in a mouse optic nerve crush (ONC) injury model. **a** Schematic of the procedure performed and the time points in which ERG recordings were taken. Vectors were administered 3 weeks prior to ONC (2 µl, 1 × 10^10^ vector particles/eye). **b** Loss of inner retinal function over time post ONC with evidence of partial improvement in pSTR responses in the AAV2 TrkB-2A-mBDNF group. **c**, **d** Preserved pSTR response compared to AAV2 Null vector injected eyes at 3 and 7 days post ONC. **e**–**g** Representative pSTRs, characteristic of RGC function, A-wave (bipolar and Müller activity), and mixed A-wave/B-wave (photoreceptor function) profiles of untreated, AAV2 TrkB-2A-mBDNF, AAV2 TrkB and AAV2 Null transduced retinas 7 days after ONC. **h** RGC (Brn3A+ cells) counts 10 days after ONC, 8 images quantified/retina. *P < 0.05, **P < 0.01 compared to AAV2 Null transduced eyes which had ONC. Control (untreated) *n* = 16, Null *n* = 11, TrkB-2A-mBDNF *n* = 14, TrkB *n* = 12. Graphs show mean ± SEM for functional readouts and mean ± SD for survival readouts with *P* values obtained via a one-way ANOVA followed by Bonferroni-modified *t*-tests for multiple comparisons

AAV2 TrkB-2A-mBDNF was then compared against a vector expressing only TrkB (AAV2 TrkB) to justify the need to upregulate both ligand and receptor for adequate protection (Fig. 7). Both vectors utilized the CAG promoter (Supplementary Figure 1a). Functional ERG recordings were also measured for each group 21 days after vector injection and again at 3 and 7 days post ONC (Fig. 7a–g and Supplementary Table 3b). Following ONC, pSTR values significantly fell to around 45% of contralateral eye readings at day 3 and then to around 35% by day 7 (*P* < 0.01; Fig. 7b–e). AAV2 TrkB showed no significant RGC functional preservation (3 days post ONC = 21.0 ± 1.4 μV, 7 days post ONC = 19.9 ± 1.7 μV, *P* = 0.49) compared to Null vector injected eyes (3 days post ONC = 19.5 ± 1.6 μV, 7 days post ONC = 16.3 ± 1.3 μV, *P* = 0.11) whereas AAV2 TrkB-2A-mBDNF demonstrated significant, partial, improvement in pSTR responses at both 3 (24.9 ± 1.7 μV, *P* < 0.05; Fig. 7b, c) and 7 days (23.4 ± 1.7 μV, *P* < 0.01; Fig. 7b, d) post ONC.

In contrast to the reduction in pSTR following ONC, there were no significant changes in negative A-wave responses and minimal impact on positive B-wave responses in any of the treated groups over time (Fig. 7f, g and Supplementary Table 3b) demonstrating a selective injury to RGCs using this injury model.

Similar to the previous experiment, following ONC, eyes injected with AAV2 Null had lost a significant proportion of their RGCs (682 ± 180 RGCs per mm^2^ vs uncrushed control, 3341 ± 384 RGCs per mm^2^, *P* < 0.001; Fig. 7h). Expressing TrkB alone was insufficient to significantly improve RGC survival (890 ± 239 RGCs per mm^2^, *P* = 0.204; Fig. 7h). AAV2 TrkB-2A-mBDNF, however, showed marked protection of RGC number (965 ± 332 RGCs per mm^2^, *P* < 0.05; Fig. 7h) demonstrating the additional benefits of improving BDNF ligand availability together with increased TrkB expression.

**Fig. 8:**
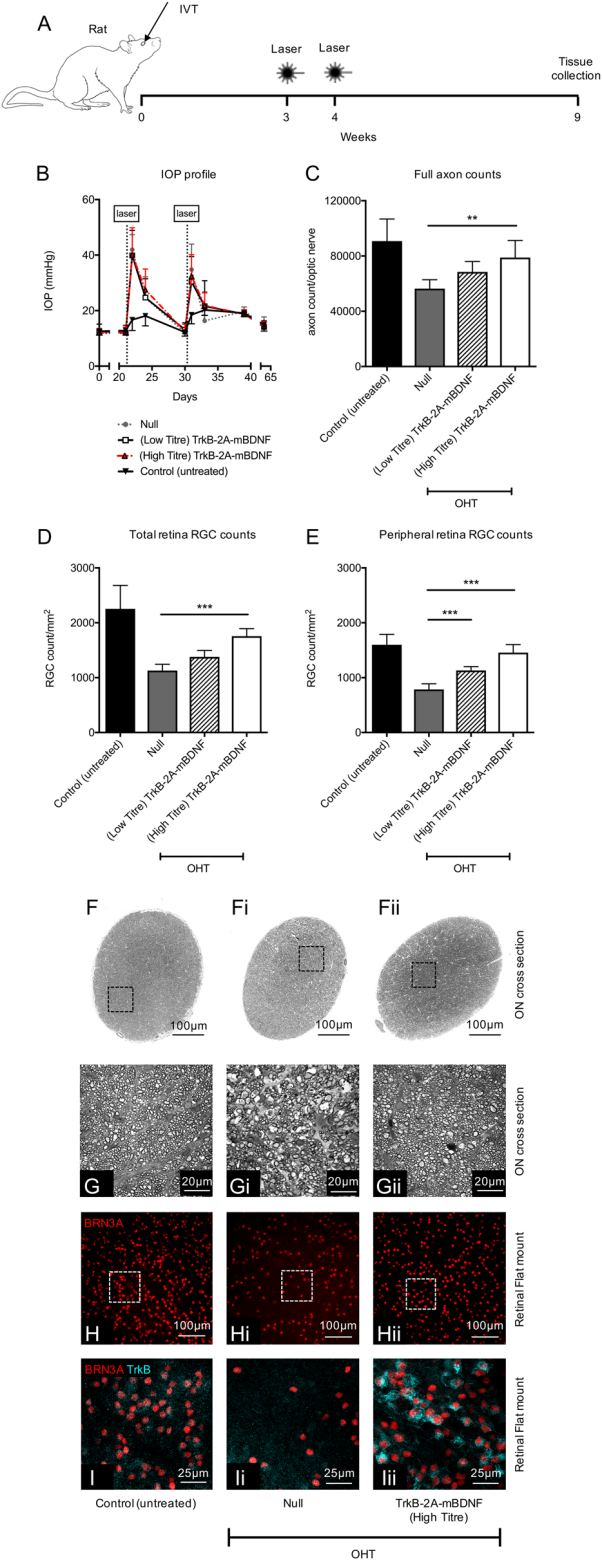
Neuroprotection using AAV2 TrkB-2A-mBDNF in rats with laser-induced ocular hypertension (OHT). **a** Schematic of the procedure performed. Vectors were administered 3 weeks prior to the first laser treatment (5 µl, 1 × 10^10^ vector particles/eye unless stated otherwise). **b** Intraocular pressure post intravitreal injection (0–21 days) and then post laser (21–65 days) across the four groups. **c** Axonal counts from within the optic nerve 6 weeks after the onset of OHT. **d** RGC (Brn3A+ cells) counts from the entire rat retinal flat mount (24 images/retina). **e** Peripheral RGC (Brn3A+ cells) counts from 16 images around the edge of the retina. **f-fii** Representative images through the optic nerve used to calculate cross-sectional area of each nerve. Black boxes (**g**–**g**ii) highlight a single region used for axon quantification. **h**–**h**ii Representative images of retinal flatmounts 6 weeks after OHT onset for each of the treatment groups. **i**–**i**ii TrkB expression on RGCs transduced with AAV2 TrkB-2A-mBDNF compared to untreated and AAV2 Null vector transduced retinas. Control (untreated) *n* = 19, Null *n* = 7, (low titre) TrkB-2A-mBDNF *n* = 7, (high titre) TrkB-2A-mBDNF *n* = 8. **P < 0.01 and ***P < 0.001 compared to AAV2 Null transduced eyes which had OHT. Low titre = 1 × 10^9^ vector particles/eye, high titre = 1 × 10^10^ vector particles/eye. Graphs show mean ± SD with *P* values obtained via a one-way ANOVA followed by Bonferroni-modified *t*-tests for multiple comparisons

Having shown AAV2 CAG TrkB-2A-mBDNF was most efficacious, the CAG vector was tested in a rat laser-induced ocular hypertension model of glaucoma to determine if protection could be demonstrated in two different species and in a model of disease as well as optic nerve injury (Fig. 8). Rats were pre-treated with a low (1 × 10^9^ VP/eye) or high dose (1 × 10^10^ VP/eye) of AAV2 CAG TrkB-2A-mBDNF and compared to eyes treated with control AAV2 CAG Null vector (1 × 10^10^ VP/eye). Vectors did not produce any changes in IOP, as measured on day 21 (Fig. 8b and previously in mice; Fig. 5b) immediately prior to laser treatment.

Trabecular laser treatment resulted in a rise in IOP to an average of 40 mmHg (Null, 40.0 ± 8.97; low titre 39.9 ± 9.03; high titre 40.1 ± 9.78 mmHg, *P* < 0.01; Fig. 8b) which returned to baseline levels within 7 days and rose again following a second laser treatment. There was no significant change in the IOP in the contralateral right eye following laser treatment. The elevated IOP rise in the treated eye then returned to baseline levels for the duration of the study (Fig. 8b). On day 63, the eyes were collected and RGCs quantified across the retina (Fig. 8d, e, h–hii).

Laser-induced IOP elevation was associated with a significant reduction in average RGC density throughout the retina compared to normotensive eyes (AAV2 Null, 1129 ± 114 RGCs per mm^2^; non-lasered control, 2253 ± 428 RGCs per mm^2^, *P* < 0.01; Fig. 8d, h–hii). Hypertensive eyes injected with AAV2 TrkB-2A-mBDNF at low or high titre showed improved RGC survival (low titre, 1379 ± 118 RGCs per mm^2^, *P* = not significant; high titre, 1757 ± 137 RGCs per mm^2^, *P* < 0.01), compared to Null vector injected eyes (Fig. 8d, h–hii). Examination of the peripheral retina demonstrated greater RGC protection, with RGC survival significantly increased with both titres (low titre, 1130 ± 71 RGCs per mm^2^, *P* < 0.001; high titre, 1456 ± 146 RGCs per mm^2^, *P* < 0.001) compared to the Null vector group (785 ± 103 RGCs per mm^2^, Fig. 8e). Brn3A staining across retinal wholemounts closely matched that of TUJ1 labeling (Supplementary Figure 3b–dii) with evidence of increased BDNF and TrkB expression in RGCs in the high titre AAV2 TrkB-2A-mBDNF group (Fig. 8i–iii and Supplementary Figure 3e–eii).

TrkB-2A-mBDNF also promoted a marked protection of optic nerve axons compared to the Null vector group, supporting the pattern of protection shown in the retina. Total RGC axon counts were reduced in IOP elevation eyes (AAV2 Null; 56,385 ± 6450 axons/ON) compared to normotensive eyes (90,800 ± 15,981 axons/ON, *P* < 0.01, Fig. 8c) but axon preservation was demonstrated when injured in the presence of AAV2 TrkB-2A-mBDNF (low titre, 68,486 ± 7482 axons/ON, *P* = not significant; high titre, 78,901 ± 12,370 axons/ON, *P* < 0.01; Fig. 8c, fi–gii).

## Discussion

Abnormalities in BDNF signaling have been reported in rodent^11–16^, dog^17^, and primate^18^ models of glaucoma. There is also increasing evidence of reduced BDNF and TrkB signaling in human glaucoma^19–22^. Several groups have demonstrated effective RGC neuroprotection via BDNF supplementation to the eye^11,12,23–27,29,44^, but this protection has been transient at least in part due to TrkB receptor downregulation^30–32,45–47^. The use of gene therapy for BDNF also poses the risk of producing accumulating levels of proBDNF, due to inadequate post-translational processing, leading to p75^NTR^ activation in retinal glia, retinal dystrophy^48^, and RGC loss^36,49^.

We recently developed a novel gene therapy construct that could overcome BDNF receptor downregulation whilst only producing the relevant mature form of BDNF, essential for RGC neuroprotection^37^. TrkB-2A-mBDNF was the best candidate identified from 20 constructs that facilitated long-term enhancement of BDNF signaling within the limited packaging size of an AAV2^37^. In the current study, we extensively tested the AAV2 TrkB-2A-mBDNF construct in vivo. We observed a stable long-term expression of both transgenes, long-term upregulation of cell survival pathways downstream of BDNF, and RGC neuroprotection following ONC in the mouse and chronic IOP elevation in the rat. In addition, we found no adverse effects of AAV2 TrkB-2A-mBDNF on retinal structure or function as assessed by histology and electroretinography.

We have previously shown that AAV2 TrkB-2A-mBDNF could be efficiently cleaved to produce both TrkB and mature BDNF within the retina^37^. Here, we quantified the expression of both proteins and showed a 3-fold increase of TrkB and mature BDNF expression compared to controls. Mature BDNF was targeted to secretory vesicles within RGCs from which the protein could be released extracellularly with the potential to act in both an autocrine and paracrine fashion (Supplementary Figure 1b). Our approach is consistent with the hypothesis that boosting BDNF release from within the retina could compensate for the reduction in BDNF retrograde transport from the brain which has been observed in glaucoma^16,18,50^. Previous animal experiments have shown AAV BDNF delivery to the superior colliculus to be ineffective in protecting RGCs in models of ONC or ocular hypertension^46^ and thus we chose intravitreal delivery as our preferred route of administration.

In addition to showing a significant elevation in mature BDNF production using our novel gene therapy, we were able to show that expression could be maintained for a minimum of 6 months following transduction and that it was only the mature, 14 kDa, BDNF being transcribed and translated. Importantly, we also demonstrated that when using the AAV2 TrkB-2A-mBDNF construct, TrkB receptors were continually expressed, avoiding the pitfalls of conventional BDNF treatments whereby BDNF overexpression is associated with TrkB receptor downregulation, limited receptor recycling, and reduced downstream signaling^30–32,46^.

We demonstrated that survival pathways involving PI3K and ERK were substantially elevated over 6 months, as others have shown after TrkB receptor activation^51,52^, with ERK signaling in particular regarded as essential for RGC survival^51,53^. This pattern of signaling pathway activation in RGCs was equivalent to effects seen in HEK293 cells transfected with plasmid DNA demonstrating a beneficial role of including both ligand and receptor within a single construct^37^. This dual approach capitalizes on previous work where upregulation of TrkB in RGCs by AAV-mediated gene transfer was shown to enhance RGC neuroprotection after optic nerve axotomy, but protection was enhanced further when combined with repeated intravitreal injections of BDNF^51^. As such, this study clearly demonstrates the benefit of overexpressing the ligand and its receptor and that this strategy might be expanded to include other ligand–receptor pairs.

It was also imperative that the construct or method of delivery did not cause any adverse effects. CAG is a constitutive non-selective promoter, and therefore would be expected to be active in all vector transduced cells, including target RGCs and some amacrine and Müller cells. However, transgene expression in non-target cells did not appear detrimental to any measure of retinal structure or function assessed. Overexpressing BDNF in Müller glia using adenovirus has been shown to extend the survival of axotomised RGCs^29^. Likewise, TrkB signaling specifically in Müller glia stimulates neuroprotection after optic nerve injury and delays retinal degeneration^54,55^ supporting the reduced importance for specific RGC targeting. We showed intravitreal injection AAV2 TrkB-2A-mBDNF caused limited gliosis and inflammation when TrkB and BDNF transgenes were maximally expressed indicating the vector were well tolerated and safe for use over extended periods. AAV2 TrkB-2A-mBDNF also did not negatively impact visual function as assessed by electroretinography in adult and aged animals.

Finally, and importantly, AAV2 TrkB-2A-mBDNF was neuroprotective in two different species and in a model of experimental glaucoma as well as after acute optic nerve injury. The novel vector outperformed conventional AAV2 BDNF^25,26,56^ (which express solely BDNF) and AAV2 TrkB, increasing our interest in this vector design as a possible treatment for glaucoma in the future.

## Conclusion

In summary, a novel recombinant AAV2 TrkB-2A-mBDNF construct has been demonstrated to mediate long-term enhancement of neuroprotective BDNF signaling with enhancement of RGC survival following ONC in the mouse and IOP elevation in the rat. No significant adverse effects on retinal structure or electrophysiological function were detected. Further studies are underway to explore the potential of this vector as a candidate for progression into clinical studies to protect RGCs in patients with glaucomatous neurodegeneration progressing towards blindness despite IOP lowering treatment.

## Electronic supplementary material

Supplementary Table 1

Supplementary Table 2

Supplementary Figure 1

Supplementary Figure 2

Supplementary Figure 3

Supplementary materials
