# Supplementary material for: Neuroprotection of retinal ganglion cells by a novel gene therapy construct that achieves sustained enhancement of brain-derived neurotrophic factor/tropomyosin-related kinase receptor-B signaling
Source: Cell Death Dis. 2018 Sep 26;9(10):1007. doi: 10.1038/s41419-018-1041-8 (PMC6158290; doi:10.1038/s41419-018-1041-8)
Supplement: Supplementary file 1 — Supplementary Table 1 [file 41419_2018_1041_MOESM1_ESM.docx]

Supplementary Table 1:

A) Primary Antibodies

| Specificity | Source | Isotype | Company | Concentration |
| --- | --- | --- | --- | --- |
| BDNF | Rabbit | Polyclonal | Santa Cruz | 1:300 (IHC)  1:200 (WB) |
| TrkB | Rabbit | Polyclonal | Abcam | 1:500 (IHC)  1:500 (WB) |
| p-TRK(Y515) | Rabbit | Polyclonal | Abcam | 1:750 (IHC)  1:750 (WB) |
| p-AKT(Ser473) | Rabbit | Polyclonal | Cell Signaling | 1:300 (IHC)  1:300 (WB) |
| t-AKT | Rabbit | Polyclonal | Cell Signaling | 1:300 (WB) |
| p-ERK1/2 (Thr202/Tyr204) | Rabbit | Monoclonal | Cell Signaling | 1:300 (IHC)  1:300 (WB) |
| ERK1/2 | Rabbit | Monoclonal | Cell Signaling | 1:100 (WB) |
| TUJ1 | Mouse | Monoclonal | Promega | 1:400 (IHC) |
| Brn3A | Goat | Polyclonal | Santa Cruz | 1:200 (IHC) |
| β-actin | Rabbit | Polyclonal | Cell Signaling | 1:1000 (WB) |
| IBA1 (AIF1) | Guinea Pig | Polyclonal | Synaptic Systems | 1:500 (IHC) |
| GFAP | Rabbit | Polyclonal | DAKO | 1:500 (IHC) |
| GFAP | Rabbit | Polyclonal | Abcam | 1:2000 (WB) |

B) Secondary Antibodies

| Specificity |  |  | Company | Concentration |
| --- | --- | --- | --- | --- |
| HRP-conjugated anti-rabbit |  |  | Vector Laboratories | 1:8000 (WB) |
| Alexa Fluor 488 goat anti-rabbit |  |  | Invitrogen | 1:1000 (IHC) |
| Alexa Fluor 555 goat anti-mouse |  |  | Invitrogen | 1:1000 (IHC) |
| Alexa Fluor 555 donkey anti-goat |  |  | Invitrogen | 1:1000 (IHC) |
| Alexa Fluor 647 goat anti-rabbit |  |  | Invitrogen | 1:1000 (IHC) |
| Alexa Fluor 647 donkey anti-rabbit |  |  | Invitrogen | 1:1000 (IHC) |
