# Supplementary material for: Neuroprotection of retinal ganglion cells by a novel gene therapy construct that achieves sustained enhancement of brain-derived neurotrophic factor/tropomyosin-related kinase receptor-B signaling
Source: Cell Death Dis. 2018 Sep 26;9(10):1007. doi: 10.1038/s41419-018-1041-8 (PMC6158290; doi:10.1038/s41419-018-1041-8)
Supplement: Supplementary file 2 — Supplementary Table 2 [file 41419_2018_1041_MOESM2_ESM.docx]

Supplementary Table 2: Electroretinogram (ERG) data for experimental cohort 4 and cohort 7

A) Cohort 4 - AAV2 BDNF long term study

| ERG recording | BDNF | TrkB-2A-mBDNF | Control (untreated) |
| --- | --- | --- | --- |
| pSTR (µV)  *20 wk:* | 35.5±1.5 | 32.5±3.7 | 35.7±0.8 |
| B-wave (µV)  *20 wk:* | 739.4±43.5 | 685.1±77.1 | 667.5±27.6 |
| A-wave (µV)  *20 wk:* | -449.8±31.1 | -492.0±65.9 | -493.6±14.4 |

Mean ± SEM

B) Cohort 7 - AAV2 TrkB-2A-mBDNF vs AAV2 TrkB optic nerve crush neuroprotection and function study

| ERG recording | TrkB | TrkB-2A-mBDNF | Null | Control (untreated) |
| --- | --- | --- | --- | --- |
| pSTR (µV)  *Pre ONC:*  *3d post ONC:*  *7d post ONC:* | 44.0±1.6  21.0±1.4  19.9±1.7 | 40.4±1.4  24.9±1.7  23.4±1.7 | 40.0±1.1  19.5±1.6  16.3±1.3 | 39.0±1.6  39.9±1.6  40.1±1.5 |
| B-wave (µV)  *Pre ONC:*  *3d post ONC:*  *7d post ONC:* | 968.3±74.6  726.3±52.0  854.10±79.0 | 814.3±32.9  793.1±79.0  872.2±79.7 | 921.9±49.8  746.6±51.7  798.6±50.6 | 985.8±62.4  866.7±42.2  913.8±32.5 |
| A-wave (µV)  *Pre ONC:*  *3d post ONC:*  *7d post ONC:* | -830.7±90.4  -880.2±117.7  -616.2±67.3 | -626.2±30.7  -610.5±46.3  -614.0±72.3 | -775.0±72.7  -600.4±64.1  -648.8±46.5 | -724.1±22.1  -765.5±74.6  -709.9±25.4 |

Mean ± SEM
