# Supplementary figures and images for: Neuroprotection of retinal ganglion cells by a novel gene therapy construct that achieves sustained enhancement of brain-derived neurotrophic factor/tropomyosin-related kinase receptor-B signaling
Source: Cell Death Dis. 2018 Sep 26;9(10):1007. doi: 10.1038/s41419-018-1041-8 (PMC6158290; doi:10.1038/s41419-018-1041-8)

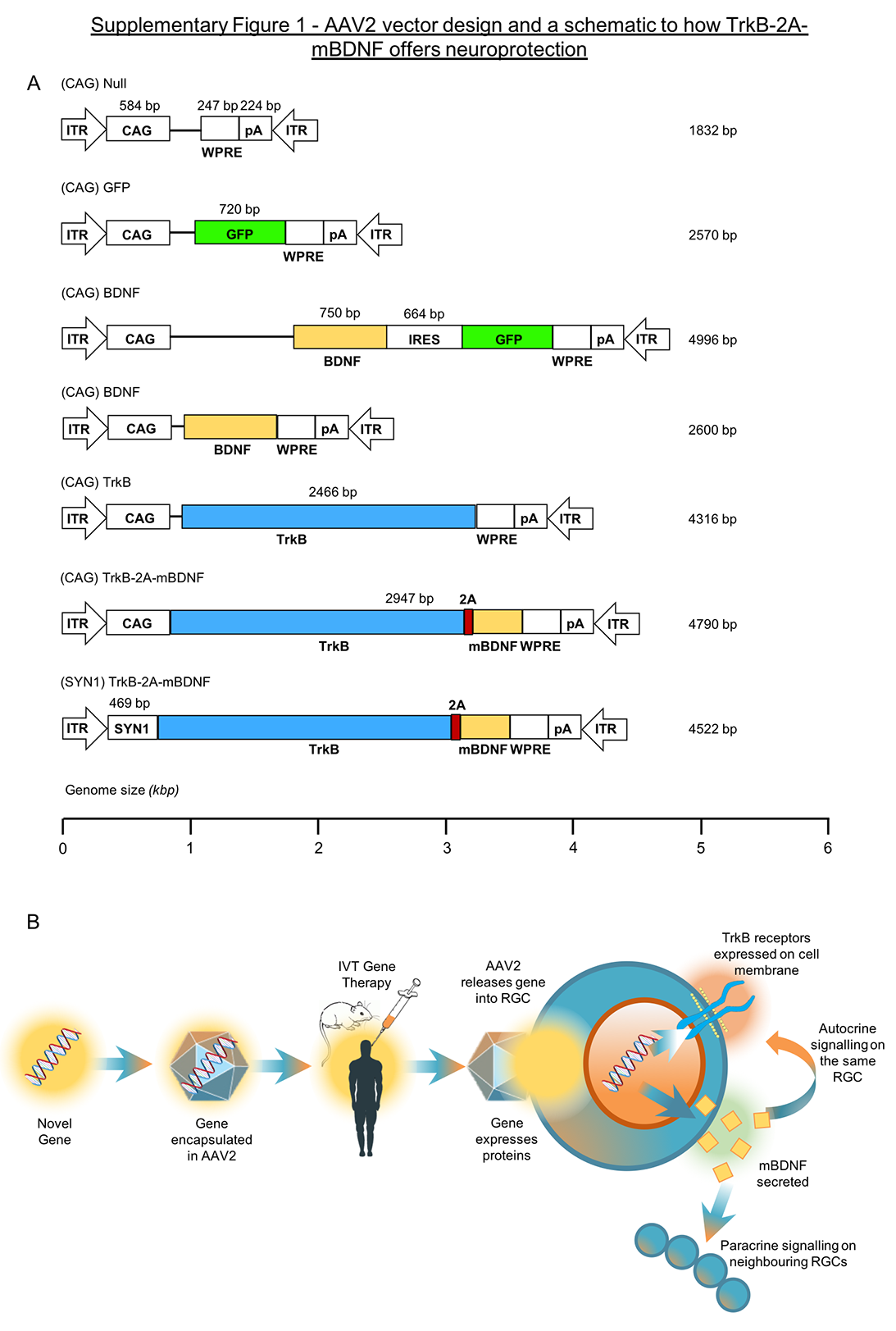

Supplement: Supplementary file 3 — Supplementary Figure 1 [file 41419_2018_1041_MOESM3_ESM.tif]

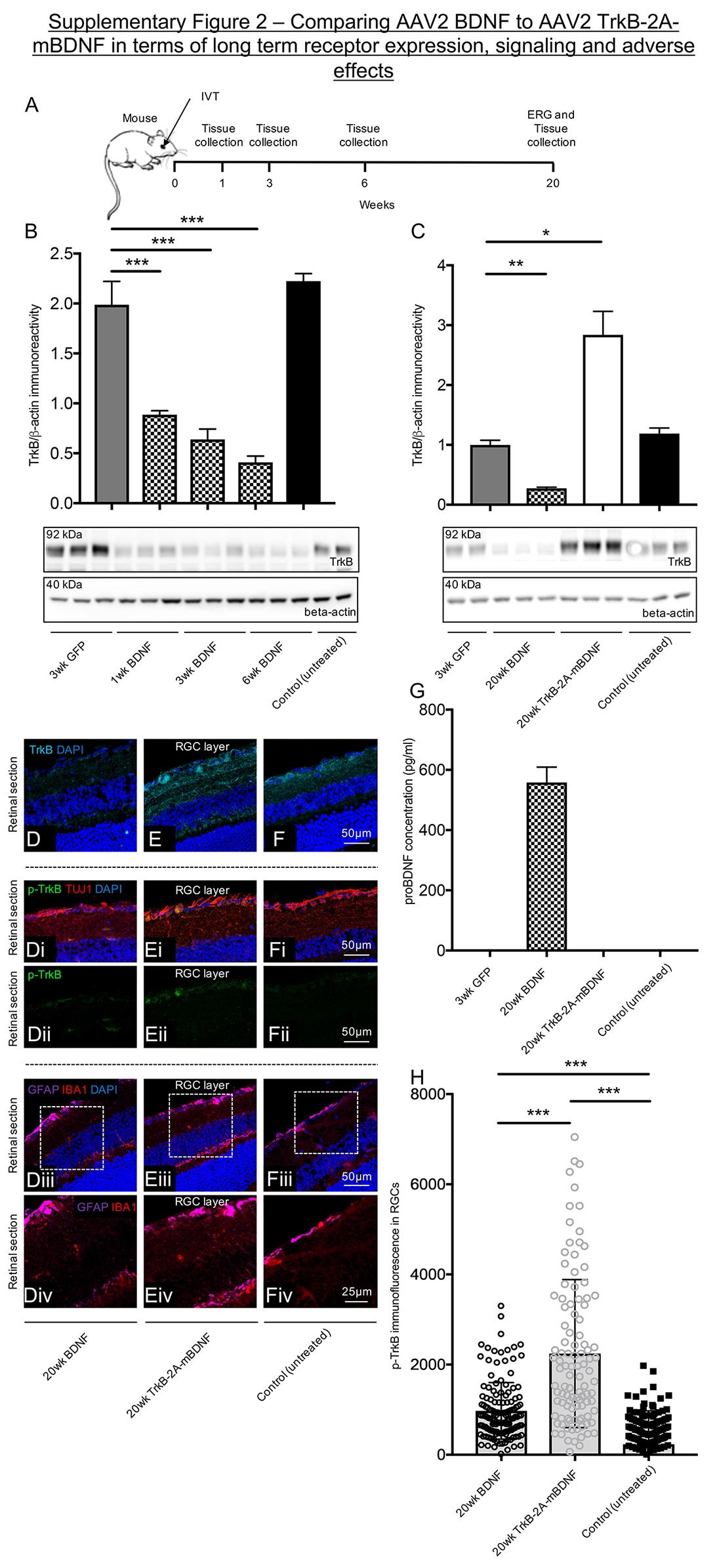

Supplement: Supplementary file 4 — Supplementary Figure 2 [file 41419_2018_1041_MOESM4_ESM.tif]

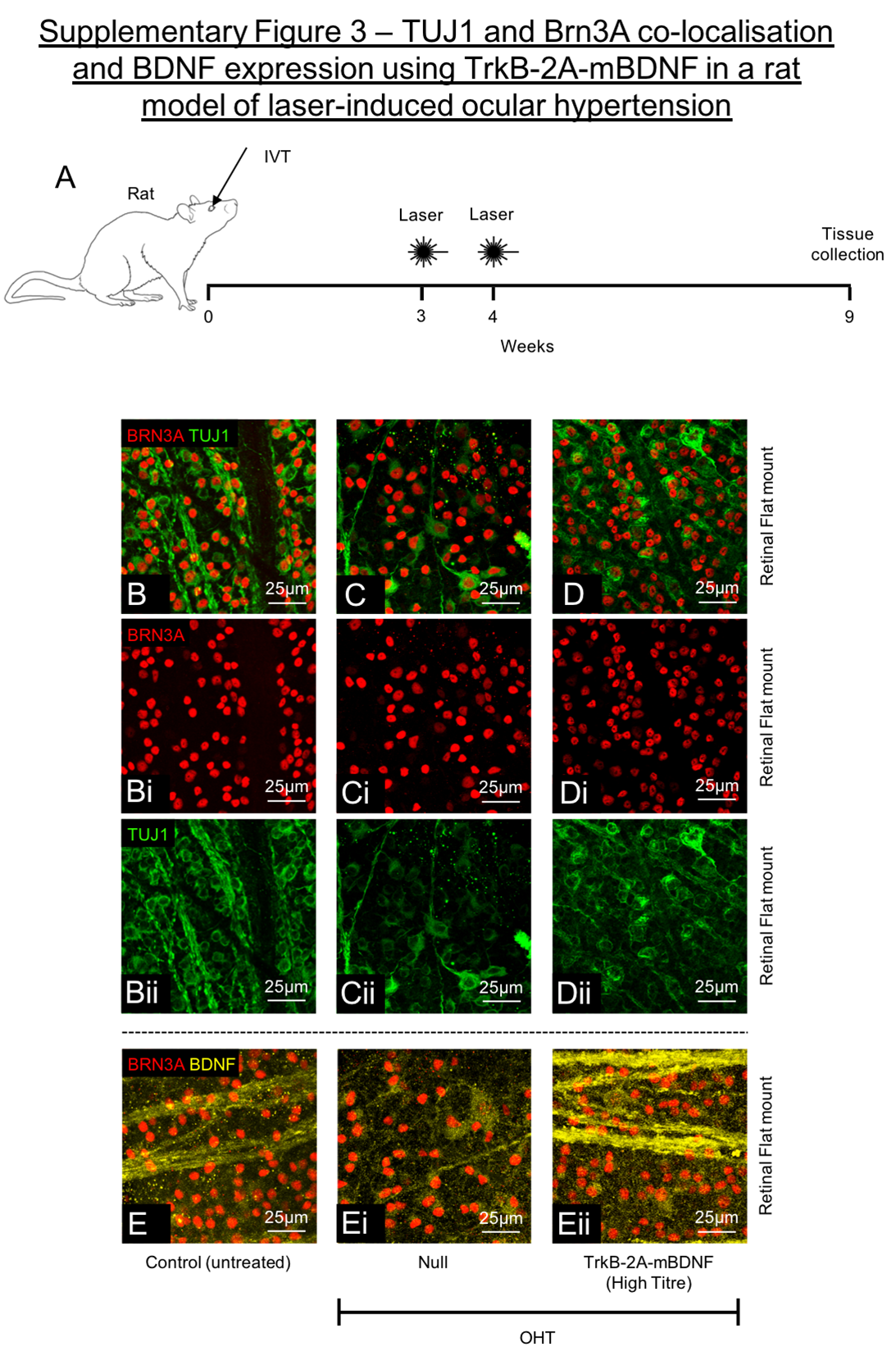

Supplement: Supplementary file 5 — Supplementary Figure 3 [file 41419_2018_1041_MOESM5_ESM.tif]
