# Supplementary material for: Neuroprotection of retinal ganglion cells by a novel gene therapy construct that achieves sustained enhancement of brain-derived neurotrophic factor/tropomyosin-related kinase receptor-B signaling
Source: Cell Death Dis. 2018 Sep 26;9(10):1007. doi: 10.1038/s41419-018-1041-8 (PMC6158290; doi:10.1038/s41419-018-1041-8)
Supplement: Supplementary file 6 — Supplementary materials [file 41419_2018_1041_MOESM6_ESM.docx]

**Supplementary Figure 1:** A) Schematic diagram of the vectors used throughout this research project. B) Schematic of how the AAV2 TrkB-2A-mBDNF vector drives the production of TrkB and BDNF to allow both paracrine and autocrine signaling on RGCs across the retina. ITR = Inverted Terminal Repeat, IRES = Internal Ribosome Entry Site, WPRE = Woodchuck Hepatitis Virus Posttranscriptional Regulatory Element, pA = Polyadenylation sequence.

**Supplementary Figure 2:** Long term vector expression and alterations in TrkB expression and phosphorylation in the mouse retina after intravitreal injection of AAV2 GFP, AAV2 BDNF or AAV2 TrkB-2A-mBDNF (2µl, 1x10^10^ vector particles/eye). A) Schematic of the procedure performed and the time points in which tissues were collected. B) Reduced TrkB expression was measured in retinal lysates shortly after AAV2 BDNF expression compared to AAV2 GFP or untreated eyes (n=3/time point). C) At 5 months, AAV2 BDNF significantly decreased TrkB expression whereas AAV2 TrkB-2A-mBDNF could increase expression over control levels (n=3/time point). D-F) Representative images of TrkB immunoreactivity in retinal sections 5 months after vector treatment (n=3). Di-Fii) Representative images of p-TrkB immunoreactivity in retinal sections 5 months after vector treatment. Diii-Fiv) Representative images of GFAP immunoreactivity and inflammatory cell marker IBA1 in retinal sections 5 months after vector treatment. G) ProBDNF expression (measured by proBDNF ELISA ^37^) in retinal lysates 5 months after vector treatment (n=3). H) p-TrkB immunofluorescence within individual RGCs (TUJ1+ cells) was significantly increased in eyes injected with AAV2 TrkB-2A-mBDNF compared to controls yet was only marginally elevated if injected with AAV2 BDNF at 5 months (n=400 RGCs from 9 retinas). * P<0.05, ** P<0.01 and *** P<0.001 compared to 3 wk GFP. Graphs show mean ± SEM with p values obtained via a one-way ANOVA followed by Bonferroni-modified t-tests for multiple comparisons.

**Supplementary Figure 3:** TUJ1 and Brn3A colocalization across the retina of rats with laser-induced ocular hypertension (OHT). A) Schematic of the procedure performed. Vectors were administered 3 weeks prior to the first laser treatment (5µl, 1x10^10^ vector particles/eye). B-Dii) TUJ1 and Brn3A showed almost identical cell labelling before and after injury with a reduction of TUJ1 positive cells after OHT in the AAV2 Null group and preservation with AAV2 TrkB-2A-mBDNF treatment. E-Eii) BDNF expression on RGCs transduced with AAV2 TrkB-2A-mBDNF compared to untreated and AAV2 Null vector transduced retinas. Control (untreated) n=19, Null n=7, (Low Titre) TrkB-2A-mBDNF n=7, (High Titre) TrkB-2A-mBDNF n=8.
